# Identification and evaluation of a potent novel ATR inhibitor, NU6027, in breast and ovarian cancer cell lines

**DOI:** 10.1038/bjc.2011.243

**Published:** 2011-07-05

**Authors:** A Peasland, L-Z Wang, E Rowling, S Kyle, T Chen, A Hopkins, W A Cliby, J Sarkaria, G Beale, R J Edmondson, N J Curtin

**Affiliations:** 1 Newcastle University, Northern Institute for Cancer Research, Newcastle upon Tyne NE2 4HH, UK; 2 Department of Radiation Oncology, Mayo Clinic, Rochester, MN 55905, USA

**Keywords:** ATR, G2 checkpoint, homologous recombination, chemosensitisation, synthetic lethality

## Abstract

**Background:**

The ataxia telangiectasia mutated and Rad3-related kinase (ATR) has a key role in the signalling of stalled replication forks and DNA damage to cell cycle checkpoints and DNA repair. It has long been recognised as an important target for cancer therapy but inhibitors have proved elusive. As NU6027, originally developed as a CDK2 inhibitor, potentiated cisplatin in a CDK2-independent manner we postulated that it may inhibit ATR.

**Methods:**

Cellular ATR kinase activity was determined by CHK1 phosphorylation in human fibroblasts with inducible dominant-negative ATR-kinase dead expression and human breast cancer MCF7 cells. Cell cycle effects and chemo- and radiopotentiation by NU6027 were determined in MCF7 cells and the role of mismatch repair and p53 was determined in isogenically matched ovarian cancer A2780 cells.

**Results:**

NU6027 is a potent inhibitor of cellular ATR activity (IC_50_=6.7 *μ*M) and enhanced hydroxyurea and cisplatin cytotoxicity in an ATR-dependent manner. NU6027 attenuated G2/M arrest following DNA damage, inhibited RAD51 focus formation and increased the cytotoxicity of the major classes of DNA-damaging anticancer cytotoxic therapy but not the antimitotic, paclitaxel. In A2780 cells sensitisation to cisplatin was greatest in cells with functional p53 and mismatch repair (MMR) and sensitisation to temozolomide was greatest in p53 mutant cells with functional MMR. Importantly, NU6027 was synthetically lethal when DNA single-strand break repair is impaired either through poly(ADP-ribose) polymerase (PARP) inhibition or defects in XRCC1.

**Conclusion:**

NU6027 inhibits ATR, impairing G2/M arrest and homologous recombination thus increasing sensitivity to DNA-damaging agents and PARP inhibitors. It provides proof of concept data for clinical development of ATR inhibitors.

The most commonly used anticancer chemotherapy and radiotherapy kill tumour cells by causing a wide variety of DNA damage. DNA is also continuously damaged by endogenous and environmental agents, with about 10 000 endogenous lesions, of which approximately 50 are DSBs, formed per cell cycle (Vilenchik and Knudson, 2003). Efficient cell cycle checkpoint signalling and DNA repair pathways have therefore evolved to preserve cell viability (Jackson and Bartek, 2009). These regulatory proteins represent exciting targets to increase the activity of chemotherapy and radiotherapy (Shrivastav *et al*, 2008).

Ataxia telangiectasia mutated and Rad3-related (ATR) is a key sensor of single-stranded DNA associated with stalled replication forks, DSB and other DNA damage repair intermediates and is critical for the progression of replication forks stalled by damaged DNA (Petermann and Caldecott, 2006). It is activated by a wide variety of DNA damage and interacts with the mismatch repair (MMR) machinery (Wang and Qin, 2003; Caporali *et al*, 2004; Yamane *et al*, 2004; Liu *et al*, 2010). It functions during the intra-S and G2/M checkpoints, via phosphorylation of CHK1, to elicit G2/M arrest (Zou and Elledge, 2003; Kastan and Bartek, 2004; O’Connell and Cimprich, 2005) and stabilises and re-starts stalled replication forks by signalling to the homologous recombination (HR) repair pathway (Chen, 2000; Wang *et al*, 2004; Sorensen *et al*, 2005). In mice, homozygous deletion of either ATR or CHK1 confers embryonic lethality, underlying the fundamental importance of this pathway (Brown and Baltimore, 2000). Genetic reduction of ATR abrogates the G2/M checkpoint and increases sensitivity to various cytotoxic anticancer agents (Cliby *et al*, 1998, 2002; Caporali *et al*, 2004; Ward *et al*, 2004; Yamane *et al*, 2004). Sensitisation is specific to replicating cells and, importantly, ATR inhibition selectively sensitised cells that are defective in the G1 checkpoint, for example, by virtue of p53 mutation (Cliby *et al*, 1998; Nghiem *et al*, 2001).

CHK1 inhibitors are being developed for clinical use (Garber, 2005; Chen *et al*, 2006; Ashwell *et al*, 2008; Dai and Grant, 2010). As CHK1 is only one of ATR's targets, inhibitors of ATR may have a different spectrum of activity. Despite the attractiveness of the target, small molecule inhibitors of ATR have proved elusive (Wagner and Kaufmann, 2010) and the progress of ATR research has been hampered by the lack of potent inhibitors. The prototype ATR inhibitor, caffeine (Supplementary Figure 1) (Sarkaria *et al*, 1999) is weak and nonspecific (Wagner and Kaufmann, 2010). The PI3K inhibitor PI-103 (Supplementary Figure 1) inhibits ATR but also inhibits ATM and DNA-dependent protein kinase (DNA-PK) (Knight *et al*, 2006), complicating evaluation of its effect on DNA damage signalling and repair.

The CDK2 inhibitor, NU2058, (Arris *et al*, 2000), profoundly sensitises cells to cisplatin cytotoxicity independently of CDK2 inhibition (Harrison *et al*, 2009) and the analogous pyrimidine, NU6027 (2,6-diamino-4-cyclohexyl-methyloxy-5-nitroso-pyrimidine; Supplementary Figure 1) has similar effects (Harrison, 2009). These observations, coupled with the known cisplatin sensitisation by ATR knockdown led us to investigate whether NU6027 inhibited ATR. We determined cellular ATR and CDK2 inhibition by NU6027, modulation of DNA damage-induced cell cycle arrest, repair by HR, enhancement of cytotoxic agents in cells with different p53 and MMR status and explored the synthetic lethality of NU6027 when DNA single-strand break repair is compromised. Our data show that NU6027 is a low micromolar inhibitor of ATR, a novel lead for further drug development and an excellent tool compound for *in vitro* studies on ATR.

## Materials and methods

### Chemicals and reagents

All chemicals and reagents were supplied by Sigma (Poole, UK), unless otherwise stated. Temozolomide, (Cancer Research UK), doxycyclin, etoposide, paclitaxel, camptothecin, the poly(ADP-ribose) polymerase (PARP) inhibitor PF-01367338 (formerly known as AG-014699, Pfizer GRD, La Jolla, CA, USA), the CHK1 inhibitor PF-00477736 (Axon MedchemBV, Groningen, The Netherlands), NU6027 and NU6252 (synthesised at Department of Chemistry, Newcastle University, UK; NU6027 is also available from Sigma) were dissolved in DMSO and stored at –20 °C. Cisplatin, dissolved in saline, and both doxorubicin and hydroxyurea, dissolved in water, were stored at –20 °C.

### Cell lines and culture

MCF7 human epithelial breast adenocarcinoma cells and L1210 murine leukaemia cells were obtained from American Type Culture Collection (Manassas, VA, USA), Chinese hamster ovary AA8 cells, and EM9 (XRCC1-defective AA8 cells) (Thompson *et al*, 1980) were a gift from Keith Caldecott, (Sussex University, UK). V-C8 (BRCA2-mutant Chinese hamster lung fibroblasts) (Kraakman-van der Zwet *et al*, 2003) and V-C8 B2 (BRCA2 reconstructed) were kindly provided by Professor Malgorzata Z Zdzienicka (Leiden University, the Netherlands). GM847KD cells are SV-40-transformed normal human fibroblasts stably transfected with doxycyclin-inducible FLAG-tagged, kinase-dead ATR. In the presence of doxycyclin, the kinase dead ATR is expressed and acts as a dominant-negative inhibitor of the native ATR (Cliby *et al*, 1998, 2002). A2780 ovarian carcinoma cells and its cisplatin-resistant derivatives, CP70-B1 and CP70-A2 (Behrens *et al*, 1987) were a gift from Robert Brown, Beatson Laboratories, Glasgow, UK); A2780 is p53 and MMR-proficient, CP70 cells lack endogenous MMR because of *hMLH1* promoter hypermethylation (Strathdee *et al*, 1999), CP70-B1 cells have functional MMR because of chromosome 3 transfer, but the CP70-A2 cells carry a transferred chromosome 3 with mutant *hMLH1* (Plumb *et al*, 2006), both CP70 derivatives carry a dominant-negative p53 mutation (Brown *et al*, 1993; Lu *et al*, 2001). All cells were cultured in RPMI 1640 medium supplemented with 10% fetal bovine serum, except the GM847 line, which was grown in Dulbecco's modified Eagle's medium. GM847KD were maintained under antibiotic selection with 400 *μ*g ml^–1^ G418 sulphate and the CP70-B1 and CP70-A2 cells were grown in 250 *μ*g ml^–1^ hygromycin B (Invitrogen, Paisley, UK). Cell lines were authenticated by the supplier, used at low passage and were mycoplasma free (MycoAlert; Lonza, Rockland, ME, USA).

### PARP activity assays

Doxycyclin, used to induce ATR-KD expression is reported to inhibit PARP activity (Alano *et al*, 2006). We therefore determined if doxycyclin inhibits PARP by measuring maximum stimulatable PARP activity in L1210 cells (as used previously to determine PARP inhibitor potency: Griffin *et al*, 1998) in the presence absence of doxycyclin (2 *μ*g ml^–1^) and in GM847KD cells pre-incubated or not with doxycyclin (2 *μ*g ml^–1^) for 48 h before assay. Briefly, cells were permeabilised with digitonin (0.15 mg ml^–1^) and subsequently exposed to blunt-ended oligonucleotide (5′-CGGAATTCCG-3′ Invitrogen) at 200 *μ*g ml^–1^ in the presence of excess NAD^+^ (7 mM) in reaction buffer (100 mM Tris-HCl, 120 mM MgCl_2_, pH=7.8) for 6 min at 27 °C. The product, PAR, was detected by immunoblot using the10H antibody (Alexander Burkle, University of Konstanz, Germany) by reference to a PAR standard curve as previously described (Plummer *et al*, 2005).

Poly(ADP-ribose) polymerase activity in exponentially growing GM847KD cells treated or not with doxycyclin (2 *μ*g ml^–1^) for 1 or 24 h, was determined by measuring *N*-methyl-*N*′-nitro-*N*-nitrosoguanidine (MNNG, 25 *μ*M)-induced cellular NAD^+^ depletion as described previously (Jacobson and Jacobson, 1997).

### Cytotoxicity assay

Cell survival was determined by exposing exponentially growing cells to cytotoxic agents and/or NU6027 for 24 h before harvesting and seeding triplicate samples for colony formation in drug-free medium. Colonies were fixed, stained with crystal violet (0.4%) and counted (Oxford Optronics Ltd, Oxford, UK) and survival expressed as a percentage of the relevant control (vehicle or NU6027 alone).

### Western blotting

Cell lysates (20–30 *μ*g protein) from exponentially growing cells were resolved on 4–20% polyacrylamide tris/glycine gels (Invitrogen) blotted onto Hybond C-membrane (GE Healthcare UK Ltd, Buckinghamshire, UK) blocked in TTBS buffer (20 mM Tris,140 mM NaCl, 0.1% (v/v) Tween-20, pH 7.6) containing 5% (w/v) non-fat dried milk and incubated overnight at 4 °C in antibody buffer (1% (w/v) non-fat dried milk in TTBS) containing primary antibody: goat anti-ATR, mouse anti-CHK1, rabbit anti-DNA-PKcs (Santa Cruz Biotech, Heidelberg, Germany); rabbit anti-CHK1^pS317^ rabbit anti-pCHK1^S345^ (Cell Signalling Tech., Danvers, MA, USA); mouse anti-(FLAG) rabbit anti-pRb^T821^ (Invitrogen); mouse anti-pRb (BD Biosciences, Oxford, UK); rabbit anti-pDNA-PKcs^S2056^ (Abcam, Cambridge, MA, USA); mouse anti-pATM^S1981^ (Millipore, Billirica, MA, USA) and mouse anti-ATM (Thermo Fisher Scientific, Waltham, MA, USA) then with peroxidase-conjugated secondary antibodies (donkey anti-goat, goat anti-mouse or goat anti-rabbit, Dako, Glostrup, Denmark) for 1 h, developed using SuperSignal West Pico Chemiluminescent Substrate (Pierce: Thermo Fisher) and quantified (Fuji LAS-3000, Raytech Scientific, Sheffield, UK).

### Flow cytometric analysis of drug-treated cells

Exponentially growing cells were exposed to cytotoxic drugs ± NU6027 as indicated in the Results section before harvesting and fixing (70% ethanol), stained with propidium iodide (CycleTest Plus DNA Reagent Kit; BD Biosciences) and analysed on a FACScan flow cytometer using the CELLQUEST programme (BD Biosciences).

### Immunofluorescent detection of DNA DSB and repair by HR (γH2AX and RAD51 focus formation)

Exponentially growing cells on coverslips were treated with PF-01367338 (10 *μ*M) for 24 h, fixed with 4% paraformaldehyde, permeabilised with 0.5% Triton-X100, 50 mM NaCl, 3 mM MgCl_2_, 300 mM sucrose and 200 mM HEPES and blocked in 10% swine serum for 1 h. They were then incubated with mouse anti-*γ*H2AX (Upstate-Millipore, Billerica, MA, USA) or rabbit anti-RAD51 (H-92, Santa Cruz Biotech) overnight at 4 °C then with Alexa Fluor 555 goat anti-rabbit IgG or Alexa Fluor 546 goat anti-mouse IgG (Invitrogen) for 1 h in darkness and mounted onto slides with VectaShield (Peterborough, UK). Images were obtained with a Leica DMR microscope and RT SE6 Slider camera Advanced Spot software version 3.408 (Diagnostic Instruments Inc., Sterling Heights, MI, USA) and analysed using Image J software (NIH public domain software).

### Statistical analysis

Data analysis was performed using GraphPad Prism (GraphPad Software, San Diego, CA, USA). Statistically significant changes were determined using two-tailed paired or unpaired Student's *t*-tests, as appropriate.

## Results

### Doxycyclin does not inhibit PARP activity

**Figure 1:**
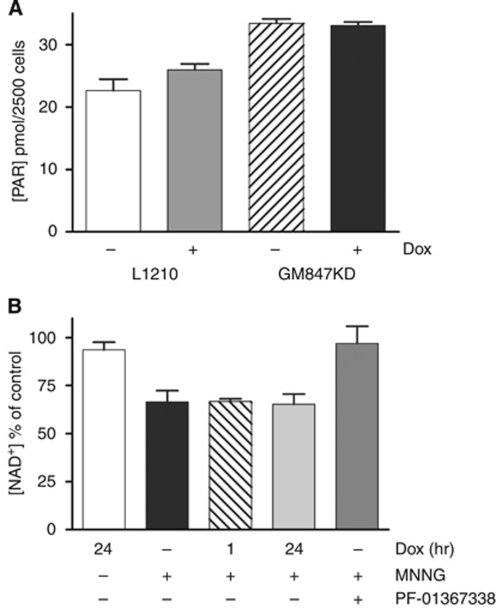
Doxycyclin does not inhibit PARP activity. (**A**) Poly(ADP-ribose) polymerase activity in L1210 cells in the presence (grey bars) or absence (white bars) of doxycyclin (2 *μ*g ml^–1^) and in GM847KD cells exposed (black bars) or not (hatched bars) to doxycyclin (2 *μ*g ml^–1^) for 48 h. Data are mean and s.e.m. of triplicate samples in a single experiment. (**B**) *N*-methyl-*N*’-nitro-*N*-nitrosoguanidine-induced NAD^+^ consumption in GM847KD cells exposed to doxycyclin (2 *μ*g ml^–1^) for 1 or 24 h before or treated for 1 h with the PARP inhibitor PF-01367338 (1 *μ*M). Data are no drug (white bars), MNNG alone (black bars) or MNNG in cells pretreated for 1 h (hatched bars) or 24 h (light grey bars) with doxycyclin (2 *μ*g ml^–1^) or 1 h with PF-01367338 (1 *μ*M, dark grey bars), mean and s.e.m. of three independent experiments.

Doxycyclin is reported to inhibit PARP activity (Alano *et al*, 2006). As doxycyclin is used to induce ATR-KD expression, and PARP is a key enzyme in DNA damage signalling and repair, this could significantly compromise analysis of the data. However, we found that doxycyclin (2 *μ*g ml^–1^) did not inhibit PARP activity in L1210 cells or in GM847KD cells pre-incubated for 48 h with doxycyclin (Figure 1A). Furthermore, exposure of GM847KD cells to doxycyclin for 1 or 24 h did not inhibit NAD^+^ depletion caused by MNNG-induced activation of PARP. In contrast, the PARP inhibitor, PF-01367338, completely inhibited MNNG-induced NAD^+^ depletion (Figure 1B).

### Phosphorylation of CHK1 at ser345 after hydroxyurea is a specific marker of ATR activity

**Figure 2:**
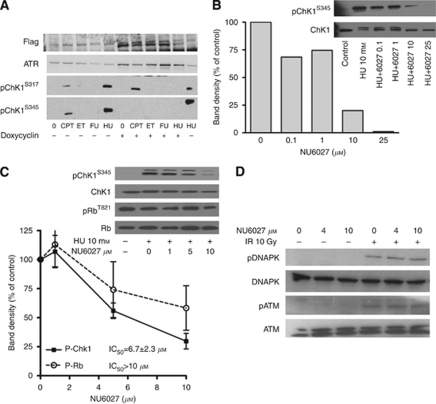
NU6027 inhibits cellular ATR activity, more potently than CDK2 and does not inhibit irradiation-induced DNA-PK or ATM activity. (**A**) Western blot for pCHK1^S317^ and pCHK1^S345^ in extracts from GM847KD cells exposed to doxycyclin (2 *μ*g ml^–1^, lanes 6–10) or not (lanes 1–5, 11) 48 h before treatment with camptothecin (CPT, 100 nM), etoposide (ET, 1 *μ*M), 5-fluorouracil (FU, 30 *μ*M) or hydroxyurea (HU, 10 mM) for 24 h. Cell extracts (20 *μ*g) were loaded per lane in duplicate gels. After transfer, the membrane was cut horizontally with the top portion incubated with anti-ATR or anti-Flag antibody and the bottom portion incubated with anti-pCHK1^S317^ or anti-pCHK1^S345^. (**B**) pCHK1^S345^ in GM847KD cells exposed to 10 mM hydroxyurea in the absence and presence of NU6027 for 24 h. Intensity of pCHK1^S345^ relative to total CHK1 is expressed as a percentage of that determined in cells treated with hydroxyurea alone. (**C**) MCF7 cells were exposed to 10 mM hydroxyurea in the absence and presence of increasing concentrations of NU6027 for 24 h. Samples were loaded onto a single gel and after cutting horizontally the top portion of the membrane was probed with anti-pRb^T821^ and the lower with anti-pCHK1^S345^ before stripping and re-probing for total Rb or CHK1 as appropriate. Intensity of pCHK1^S345^ was measured as described above. Similarly, CDK2-specific pRb phosphorylation at T821 normalised to total pRb is expressed as a percentage of that determined in cells not exposed to NU6027. Data are mean and bars are s.e.m. of 3 independent experiments. (**D**) Western blot of DNA-PK and ATM and irradiation-induced autophosphorylation in extracts from MCF7 cells pre-exposed or not to NU6027 at 4 or 10 *μ*M for 2 h before and 1 h after irradiation (10 Gy).

CHK1 is phosphorylated by ATR at positions ser 317 and 345, however, these may overlap with ATM-mediated phosphorylation events. We therefore measured CHK1 phosphorylation induced by DNA-damaging agents in GM847KD with and without dominant-negative ATR-KD expression. Flag and ATR expression were increased following doxycyclin treatment, confirming ATR-KD induction (Figure 2A). Camptothecin and hydroxyurea, but not etoposide and 5-fluorouracil, induced robust phosphorylation of CHK1. Hydroxyurea-induced phosphorylation at both sites was inhibited when ATR-KD was activated but only ser345 phosphorylation was inhibited in the camptothecin-treated cells. These data demonstrate that pCHK1^S345^ is specific for ATR and that camptothecin-induced DNA damage may activate ATM to phosphorylate pCHK1 on Ser317 but hydroxyurea-mediated damage is signalled to CHK1 only via ATR.

### NU6027 is a more potent inhibitor of ATR than CDK2 in intact cells and does not inhibit cellular DNA-PK or ATM

Using pCHK1^S345^ as a marker, we measured ATR inhibition by NU6027 in GM847KD and human breast cancer, MCF7 cells (Figures 2B and C). In GM847KD cells, the IC_50_ was 2.8 *μ*M (single experiment) and in MCF7 cells it was 6.7±2.3 *μ*M (mean±s.d. of three independent experiments). NU6027 was a less potent inhibitor of CDK2 and 10 *μ*M NU6027 inhibited CDK2-mediated pRb^T821^ by 42±27% compared with 70±12% inhibition of pCHK1^S345^ (mean±s.d. of three independent experiments). In cell-free biochemical assays, the IC_50_ of NU6027 against CDK2 is 2.2 *μ*M (Arris *et al*, 2000) and the Ki against ATR is 100 nM (evaluated as described by Charrier *et al*, 2011: single estimation, John Pollard, Vertex Pharmaceuticals, personal communication). To confirm that NU6027 chemo and radiosensitisation was not due to inhibition of ATM or DNA-PK, we exposed MCF7 cells to IR (10 Gy) in the presence or absence of NU6027. NU6027 (2 h pre-exposure and 1 h post IR exposure to 4 or 10 *μ*M) did not inhibit IR-induced autophosphorylation of DNA-PK (pDNA-PKcs^S2056^) or ATM (pATM^S1981^) (Figure 2D).

### NU6027 sensitises cells to DNA-damaging therapy by inhibiting ATR

**Figure 3:**
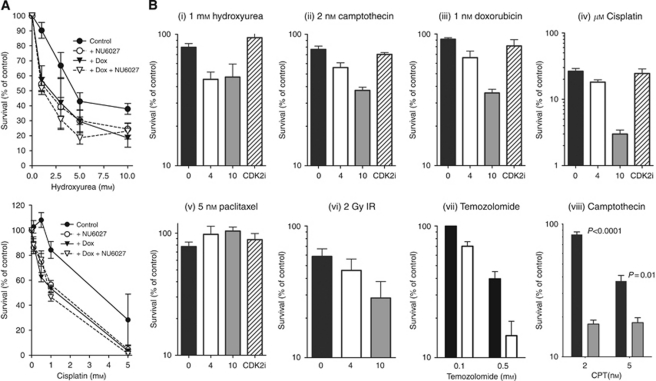
Chemo- and radiosensitisation by NU6027. (**A**) NU6027 potentiation of hydroxyurea and cisplatin is dependent on ATR activity. Clonogenic survival of GM847KD cells, with (triangles) or without (circles) 48-h exposure to doxycyclin and exposed to increasing concentrations of hydroxyurea or cisplatin in the presence (open symbols) or absence (filled symbols) of 4 *μ*M NU6027 for 24 h. Data, normalised to DMSO control or NU6027 alone control as appropriate, are mean and s.e.m. of 3 independent experiments. (**B**) NU6027 decreases the survival of MCF7 cells exposed to various DNA-damaging agents but not an antitubulin agent. Clonogenic survival of cells exposed for 24 h to cytotoxic agent alone (black bars) or in the presence of 4 *μ*M NU6027 (white bars) or 10 *μ*M NU6027 (grey bars) or 10 *μ*M NU6252 (CDK2i; hatched bars) to control for CDK2-mediated effects. Data, normalised to DMSO control or NU6027 alone control as appropriate, are mean and s.e.m. of three independent experiments (i–vi) or mean of three replicates in two independent experiments (vii and viii) normalised to vehicle alone, NU6027 or NU6252 alone controls.

We used GM847KD cells to verify that chemosensitisation by NU6027 was ATR-dependent. GM847KD cells were significantly more sensitive to both hydroxyurea (2.5-fold; *P*=0.006) and cisplatin (1.8-fold; *P*=0.002) following ATR-KD induction by doxycyclin (Figure 3A and Table 1). NU6027 significantly potentiated hydroxyurea (3.2-fold; *P*=0.005) and cisplatin (2-fold; *P*=0.02) in the absence, but not in the presence of doxycyclin.

### NU6027 sensitises human breast cancer cells to a wide range of DNA-damaging cytotoxic drugs but not an antitubulin agent

We investigated chemosensitisation of the major classes of anticancer drugs by NU6027 in MCF7 cells. Cells were exposed to a single concentration of cisplatin (a DNA cross-linking agent), hydroxyurea (an antimetabolite), camptothecin (a topoisomerase I poison), doxorubicin (a topoisomerase II poison) and paclitaxel (an antitubulin agent) in the presence or absence of 4 or 10 *μ*M NU6027 (Figures 3Bi–v). To eliminate CDK2 inhibition as a cause of chemosensitisation, we investigated chemosensitisation by a potent CDK2 inhibitor, NU6252 (10 *μ*M; cell-free IC_50_=19 nM) that does not inhibit ATR (Supplementary Figure 2). NU6027 was not cytotoxic at 4 *μ*M (survival >90%) and only mildly cytotoxic at 10 *μ*M (survival >75%). As expected, NU6027 (but not NU6252) significantly potentiated cisplatin (1.4-fold; *P*=0.059 at 4 *μ*M and 8.7-fold; *P*=0.0012 at 10 *μ*M), doxorubicin (1.3-fold; *P*=0.0079 at 4 *μ*M and 2.5-fold; *P*<0.0001 at 10 *μ*M), camptothecin (1.4-fold; *P*=0.0223 at 4 *μ*M and 2-fold; *P*=0.018 at 10 *μ*M) and hydroxyurea (1.8-fold; *P*=0.0147 at 4 *μ*M and 0.0412 at 10 *μ*M). Predictably, NU6027 failed to have any impact on paclitaxel-induced cytotoxicity (Figure 3Bv). NU6027 also potentiated 2 Gy IR in a concentration-dependent manner (Figure 3Bvi, 1.4-fold; *P*=0.0126 at 10 *μ*M). Potentiation was not related to the degree of cytotoxicity of the primary agent; NU6027 potentiated the cytotoxicity of camptothecin and temozolomide (a DNA methylating agent) at concentrations above and below their LC_50_ (Figure 3Bvii, 1.8-fold; *P*=0.0008 at 0.1 mM temozolomide and 2.7-fold; *P*=0.0041 at 0.5 mM temozolomide, Figure 3Bviii, 4.7-fold; *P*<0.0001 at 2 nM camptothecin and 2-fold at 5 nM camptothecin *P*=0.0114). In contrast, the CHK1 inhibitor PF-00477736 at 360 nM as previously described (Blasina *et al*, 2008) was more cytotoxic in its own right (survival=45%) and caused a 1.6-fold sensitisation of cisplatin (*P*=0.008) but no significant sensitisation of camptothecin or paclitaxel, in contrast to the previously described disruption of the mitotic checkpoint as well as the DNA damage checkpoint (Zhang *et al*, 2009) (Supplementary Figure 3).

### NU6027 enhancement of cisplatin and, temozolomide cytotoxicity and attenuation of G2/M arrest in cells with different p53 and MMR status

**Figure 4:**
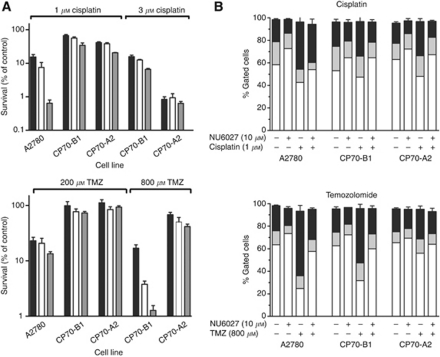
Chemosensitisation and cell cycle effects of NU6027 in ovarian cancer cells with differing p53 and MMR status. (**A**) Cytotoxicity of cisplatin (top panel), temozolomide (bottom panel) alone (black bars) or in the presence of 4 *μ*M NU6027 (white bars) or 10 *μ*M NU6027 (grey bars). A2780, CP70-B1 and CP70-A2 cells were treated for 24 h. Data, normalised to DMSO control or NU6027 alone control as appropriate, are mean and s.e.m. of three independent colony-formation experiments. (**B**) Cell cycle distribution of A2780, CP70-B1 and CP70-A2 cells treated with cisplatin 24 h (top panel), temozolomide 24 h (bottom panel) alone or in the presence of 10 *μ*M NU6027 as indicated. Data are G1 (white bars), S (grey bars) and G2/M (black bars), mean and s.e.m. of three independent experiments.

Functional MMR is necessary for the signalling of temozolomide-induced DNA damage. Mismatch repair-defective cells are resistant to temozolomide and cisplatin. We therefore investigated the effect of NU6027 on cell cycle arrest and cytotoxicity of these agents in human ovarian cancer cells with differing MMR and p53 status; A2780 (MMR+ and p53+), CP70-B1 (MMR+, p53–) and CP70-A2 (MMR–, p53–) (Figure 4). NU6027 caused a modest G1 arrest in all cells (presumably by virtue of CDK2 inhibition) and was not cytotoxic *per se*. As expected, cisplatin was much more cytotoxic to the A2780 cells. Cisplatin-induced G2/M arrest was more pronounced in A2780 cells, in line with its greater cytotoxicity. NU6027 attenuated G2/M arrest and, at 10 *μ*M, caused significant chemosensitisation of all of the cell lines. Chemosensitisation was greatest in the A2780 cells (20-fold; *P*=0.012) compared with only 2-fold in the CP70-B1 and CP70-A2 cells (*P*=0.027 and 0.016, respectively) and A2780 cells were the only ones to be significantly (*P*=0.029) sensitised by 4 *μ*M NU6027.

A2780 cells were sensitive to temozolomide (200 *μ*M) and although NU6027 (10 *μ*M) caused a further 50% reduction in survival, this was not statistically significant. The higher concentration of temozolomide (800 *μ*M) killed all A2780 cells but around 15% of CP70-B1 and 60% CP70-A2 cells survived, indicating that both p53 and MMR have significant roles in temozolomide-induced cell death. NU6027 caused profound and significant enhancement of temozolomide cytotoxicity in CP70-B1 cells at both 4 and 10 *μ*M (reduction in survival=76%, *P*=0.0098, and 93%, *P*=0.0076, respectively) but in CP70-A2 cells only 10 *μ*M caused a modest but significant enhancement (approximately 40%, *P*=0.0351). Temozolomide caused a profound G2/M arrest in A2780 cells and to a slightly lesser extent in CP70-B1 cells, which was almost totally reversed by NU6027.

### NU6027 attenuates G2/M arrest, inhibits HR function and is synthetically lethal in combination with PARP inhibition

**Figure 5:**
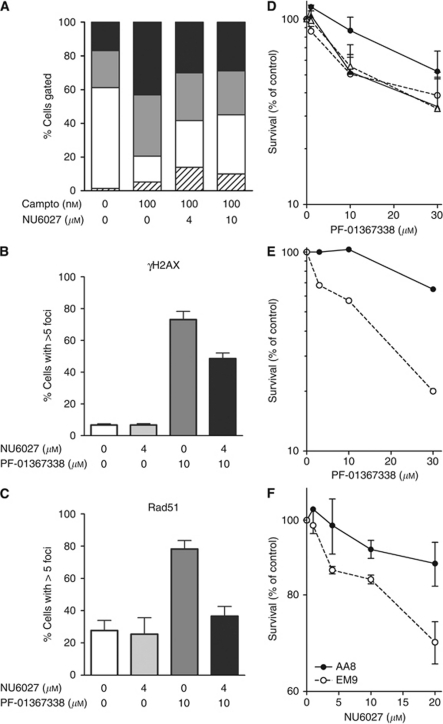
NU6027 attenuated G2/M arrest and inhibits HR in MCF7 cells and is synthetically lethal with a PARP inhibitor and in XRCC1-defective cells. (**A**) NU6027 attenuates G2/M arrest in MCF7 cells. Cell cycle distribution following 24-h exposure to camptothecin (100 nM) in the presence or absence of 4 or 10 *μ*M NU6027, as indicated. Data are sub-G1 (hatched bars), G1 (white bars), S (grey bars) and G2/M (black bars). (**B**) NU6027 mildly inhibits induction of DNA double-strand break by PF-01367338. Cells with > 5 *γ*H2AX foci following exposure to no drug (white bars) NU6027 (4 *μ*M) alone (pale grey bars) the PARP inhibitor PF-01367338 (10 *μ*M) alone (dark grey bars), or the combination (black bars). Data are mean and s.d. of three independent experiments. (**C**) NU6027 profoundly inhibits HR. Cells with > 5 RAD51 foci following exposure to no drug (white bars) NU6027 (4 *μ*M) alone (pale grey bars) the PARP inhibitor PF-01367338 (10 *μ*M) alone (dark grey bars), or the combination (black bars). Data are mean and s.d. of three independent experiments. (**D**) NU6027 potentiates PF-01367338 cytotoxicity in an ATR-dependent manner. Survival of GM847KD cells with (triangles) or without (circles) 48-h doxycyclin induction of ATR-KD, exposed to increasing concentrations of PF-01367338 in the presence (open symbols) or absence (filled symbols) of 4 *μ*M NU6027 for 24 h. Data are mean and s.e.m. of >3 independent experiments. (**E**) NU6027 potentiates PF-01367338 cytotoxicity in MCF7 cells. Survival of MCF7 cells exposed to increasing concentrations of PF-01367338 in the absence (filled circles, solid line) or presence (open circles, broken line) of 4 *μ*M NU6027 for 24 h. Data, normalised to DMSO control or NU6027 alone control as appropriate, are mean of duplicate samples in a single representative experiment, of which two were conducted. (**F**) NU6027 is more cytotoxic to cells lacking XRCC1. Survival of AA8 (filled circles) and EM9 (open circles) cells exposed to increasing concentrations of NU6027 for 24 h before seeding for colony formation. Data are mean and s.e.m. of triplicate evaluations in two independent experiments.

We investigated inhibition of G2/M arrest and HR by NU6027 after cytotoxic exposure in MCF7 cells. Camptothecin caused profound G2/M arrest, with an increase in the G2/M fraction from 17±2% to 43±6% (s.d.) (Figure 5A). This arrest was significantly inhibited by NU6027 to 29±2% (s.d.). RAD51 focus formation after 24-h exposure to the PARP inhibitor, PF-01367338, is a reliable indicator of HR function (Mukhopadhyay *et al*, 2010). We confirmed this in mammalian cells deficient (V-C8) and proficient (V-C8 B2) in BRCA2, an essential component of HR. PF-01367338 caused an approximately three-fold increase in RAD51 foci in the V-C8 B2, but not the V-C8 cells and NU6027 significantly reduced RAD51 foci in both control and PF-01367338-treated V-C8 B2 cells (Supplementary Figure 4). Inhibition of the PARP-dependent repair of endogenous DNA single-strand breaks by PF-01367338 leads to their conversion to double-strand breaks/stalled replication forks during S-phase, triggering *γ*-H2AX focus formation. In MCF7 cells, PF-01367338 caused a 10-fold increase in *γ*-H2AX foci-positive cells (Figure 5B). This was accompanied by a three-fold increase in cells with >5 RAD51 foci, indicative of HR (Figure 5C). NU6027 had no effect on either *γ*-H2AX or RAD51 foci in control cells but in PF-01367338-treated cells NU6027 caused a profound, 82%, and significant (*P*=0.0008) suppression of the increase in RAD51 foci-positive cells (Figure 5C). Indeed, following exposure to both drugs the number of RAD51-positive cells was not significantly different from baseline levels. NU6027 also caused a modest 36% reduction in *γ*-H2AX foci-positive cells, such that there was still a very significantly (*P*<0.0001) higher level than baseline (Figure 5B). We believe this may be due to CDK2 inhibition leading to fewer cells entering S-phase. The much greater effect on RAD51 than *γ*-H2AX suggest that NU6027 compromises HR.

Poly(ADP-ribose) polymerase inhibitors are synthetically lethal in cells lacking HR (Bryant *et al*, 2005), so we investigated if NU6027 and PF-01367338 exhibited synergistic cytotoxicity in GM847KD cells (Figure 5D, Table 1). The LC_50_ of PF-01367338 was reduced from >30 to 12 *μ*M following ATR-KD induction. NU6027 sensitised the ATR active cells to the same extent (LC_50_=11 *μ*M) but produced no further sensitisation in cells when ATR-KD was induced. Thus, NU6027 is synthetically lethal to PARP-inhibited cells in an ATR-dependent manner. NU6027 also substantially increased PF-01367338 cytotoxicity in MCF7 cells (Figure 5E). The XRCC1-defective EM9 cells (lacking DNA single-strand break repair) were also significantly more sensitive to NU6027 cytotoxicity than their repair-competent parental AA8 cells (Figure 5F).

## Discussion

Genetic knockdown of ATR indicated that a small molecule ATR inhibitor would be useful as radio- and chemosensitisers. Despite the identification of caffeine as a weak ATR inhibitor over 10 years ago (Sarkaria *et al*, 1999), until recently there were no novel small molecules reported to inhibit ATR. This partially stems from the difficulty in establishing an appropriate high throughput *in vitro* system, which would presumably also require ATRIP and other components (Wagner and Kaufmann, 2010). Recently, however, Vertex Pharmaceuticals (San Diego, CA, USA) have discovered a potent ATR inhibitor (Ki=6 nM), which inhibits the phosphorylation of a target peptide by purified ATR, although the details of the assay are not revealed (Charrier *et al*, 2011). This compound, VE-821, also blocked phosphorylation of CHK1 at ser345 (Reaper *et al*, 2011). We have taken a different approach, based on the observation that NU2058 and NU6027 sensitised cells to cisplatin independently of their CDK2 inhibitory activity (Harrison, 2009; Harrison *et al*, 2009). We developed a cell-based assay using ATR kinase-dead cells, and used it to demonstrate that NU6027 is, in fact, a more potent inhibitor of ATR than CDK2 in living cells. The specificity of kinase inhibitors is likely always to be an issue, for instance the effects of LY294002 are frequently still attributed to its activity against PI3K (e.g., Zhang *et al*, 2010), even when these involve radiosensitivity (Kimple *et al*, 2010), when LY294002 has long been known to be an equally potent inhibitor of DNA-PK (Izzard *et al*, 1999). We acknowledge that NU6027 also inhibits CDK2 but have confirmed that chemosensitisation is due to ATR inhibition by the use of ATR-KD cells. Furthermore another, more potent, CDK2 inhibitor that does not inhibit ATR did not potentiate the DNA-damaging agents’ cytotoxicity.

We found that NU6027 increased the cytotoxicity of all classes of DNA-damaging agents used in the treatment of cancer: DNA methylating agents (temozolomide), DNA cross-linking agents (cisplatin), topoisomerase I and II poisons (camptothecin, doxorubicin), antimetabolites (hydroxyurea) and ionising radiation. This was not due to nonspecific chemosensitisation because NU6027 did not enhance the antitubulin agent, paclitaxel, which does not damage DNA. Similarly, the recently identified inhibitor VE-821 enhanced the cytotoxicity of cisplatin, gemcitabine, camptothecin etoposide and IR, but not docetaxel in HCT 116 colon cancer cells (Reaper *et al*, 2011). The spectrum of NU6027 activity is clearly different from that of the CHK1 inhibitor, PF-00477736 (Supplementary Figure 3 and Zhang *et al*, 2009). Several CHK1 inhibitors, with different potencies, specificities and activities are currently undergoing clinical trial (reviewed in Ma *et al*, 2011) and ATR inhibition is likely to have different therapeutic activity compared with the CHK1 inhibitors.

NU6027 profoundly chemosensitised MCF7 cells, which have wild-type p53 and NU6027 caused a more profound sensitisation of cisplatin in p53 wild-type A2780 cells than p53 mutant CP70-B1 and CP70-A2 cells. We also noted potent chemosensitisation in GM847KD cells, which are SV40 transformed and so lack p53 function. These data do not support the hypothesis that ATR inhibitors should selectively chemosensitise cells with p53 dysfunction. Similarly, the CHK1 inhibitors are active in cells with functional p53 and selectivity for p53 mutant cells may be schedule-dependent (reviewed in Ma *et al*, 2011). Aberrant G1 control is not solely attributable to p53 but seems to be so common as to be a general characteristic of cancerous cells (Massague, 2004). Selective sensitisation of cells with G1 checkpoint dysfunction by ATR inhibitors may therefore only be seen when comparing normal and cancerous cells. NU6027 does not have sufficient aqueous solubility for *in vivo* evaluation to address this question but its promising cellular activity provides a sound basis for the development of further inhibitors for more advanced pre-clinical investigations.

NU6027 enhanced the cytotoxicity of temozolomide to a greater extent in CP70-B1 cells than CP70-A2 cells but increased cisplatin cytotoxicity to the same extent. Thus, functional MMR appears to be necessary for temozolomide but not cisplatin sensitisation by an ATR inhibitor. Similarly, ATR activation by BCNU (which, like cisplatin, causes DNA cross-links), was found to be independent of MMR status but temozolomide only activated ATR in MMR competent cells (Cui *et al*, 2009). These data imply that the roles of p53 and MMR in the sensitisation by ATR inhibition are complex and may be both cell-line and cytotoxic agent dependent.

**Figure 6:**
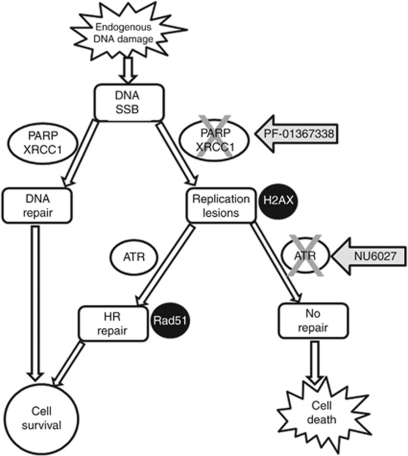
Proposed model for the synthetic lethality of ATR inhibition in cells with inactivated PARP or XRCC1. Endogenously generated DNA damage, largely in the form of single-strand breaks or base damage that is processed to single-strand breaks, are repaired by PARP and XRCC1 to promote cell survival. Unrepaired breaks accumulate in the absence of PARP or XRCC1 activity leading to stalled replication forks or replication-associated double-strand breaks (detected by *γ*H2AX foci), which activate ATR to promotes repair by HR (detected by Rad51 foci) and cell survival. If ATR is inhibited by NU6027, the replication lesions remain unrepaired and the cell dies.

Most excitingly, we found that NU6027 was synthetically lethal with PARP inhibition and XRCC1 defects. We propose this is due to its negative impact on HR, as demonstrated by the ablation of RAD51 focus induction, combined with the well-established synthetic lethality of PARP inhibitors in cells with HR defects (Reinhardt *et al*, 2009). We propose (Figure 6) that endogenously generated DNA single-strand breaks go unrepaired in the absence of PARP or XRCC1, leading to replication lesions that activate ATR to promote repair by HR. When ATR is inhibited the lesions persist and cell death ensues. Polymorphisms in XRCC1 are associated with cancer (Kiyohara *et al*, 2006) and this may be exploitable by ATR inhibition. Other defects in DNA single-strand break repair, for example, those due to aberration in DNA polβ are also associated with cancer (Starcevic *et al*, 2004). Recent data demonstrate that caffeine selectively radiosensitises polβ-defective cells (Neijenhuis *et al*, 2010) implicating that ATR inhibition would have broad applicability in cancer. Furthermore, it is well recognised that oncogene activation itself causes stalled/collapsed replication forks, making such cancer cells particularly dependent on ATR for survival (reviewed in Halazonetis *et al*, 2008) further supporting the notion that ATR inhibitors will be tumour specific.

In conclusion, we have identified NU6027 as a low micromolar inhibitor of ATR activity in human cells. NU6027 sensitises cells to the major classes of DNA-damaging anticancer therapeutics. Importantly, NU6027 is synthetically lethal when DNA single-strand break repair is impaired either through PARP inhibition or defects in XRCC1. NU6027 represents an important advance in ATR research and will be a useful tool for further studies. Clearly, further investigations are needed to determine tumour selectivity and the safety of ATR inhibitors alone and in combination with DNA-damaging agents before clinical investigations can be contemplated. Nevertheless, these data presented here provide the proof of concept groundwork for subsequent development of ATR inhibitors with the desired pharmacological properties.

## Figures and Tables

**Table 1:** Effect of activation of kinase dead ATR in GM847KD cells and co-incubation with NU6027 on the cytotoxicity of cisplatin and hydroxyurea and synthetic lethality with PARP inhibition

	**LC_50_**		
	**Hydroxyurea (mM)**	**Cisplatin (*μ*M)**	**PF-01367338 (*μ*M)**
Control	4.8±1.0	2.65±0.32	>30
+Doxycyclin	1.9±0.8	1.49±0.48	12.5±8.6
+NU6027	1.5±0.5	1.34±0.24	13.3±10.4
+Doxycyclin+NU6027	1.2±0.2	0.99±0.14	12.3±8.8

Abbreviations: ATR=ataxia telangiectasia mutated and Rad3-related; PARP=poly(ADP-ribose) polymerase.

Data are mean±s.d. of LC_50_ values calculated from at least three independent experiments as shown in Figures 2D and 5D.
