# Supplementary material for: Identification and evaluation of a potent novel ATR inhibitor, NU6027, in breast and ovarian cancer cell lines
Source: Br J Cancer. 2011 Jul 5;105(3):372–81. doi: 10.1038/bjc.2011.243 (PMC3172902; doi:10.1038/bjc.2011.243)

**Supplementary Figures**

**Figure 1. Chemical structure of NU6027 in comparison with caffeine, theophylline and PI-103**

Ki values determined by biochemical assay as described by Sarkaria et al 1999 and Kinght et al 2006

**Figure 2. NU6252 does not inhibit ATR**

Western blot for pCHK1^S317^ and pCHK1^S345^ in extracts from untreated MCF7 cells (lane 1) or MCF7 cells exposed to 10 mM hydroxyurea for 24 hr (lanes 2-4) in the presence and absence of NU6252 (lane 3) and NU6027 (lane 4).

**Figure 3. Chemosensitisation of MCF7 cells by PF-00477736**

Survival of cells exposed for 24 hr to cytotoxic agent alone (black bars) or in the presence of 360 nM PF-00477736 (white bars). Data are mean and SD of 3 replicates normalised to vehicle alone or PF-00477736 alone controls, * indicates significant difference from cytotoxic alone.

**Figure 4. Effect of NU6027 on Rad51 focus formation induced by PF-01367338 in cells with mutant BRCA2 (V-C8) or corrected BRCA2 (V-C8-B2).**

Cells were exposed to control medium (filled circles), 4 µM NU6027 (open circles), 10 µM PF-01367338 (filled triangles) or the combination of 4 µM NU6027 and 10 µM PF-01367338 (open triangles) for 24 hr prior to fixing, staining and counting of RAD51 foci, as described in the Methods. Data points are individual nuclei. PF-01367338 caused a significant elevation of Rad51 foci in comparison to control, which was significantly inhibited by NU6027 in the BRCA2 corrected cells only, as indicated in the figure.

**Supplementary Figure 1**


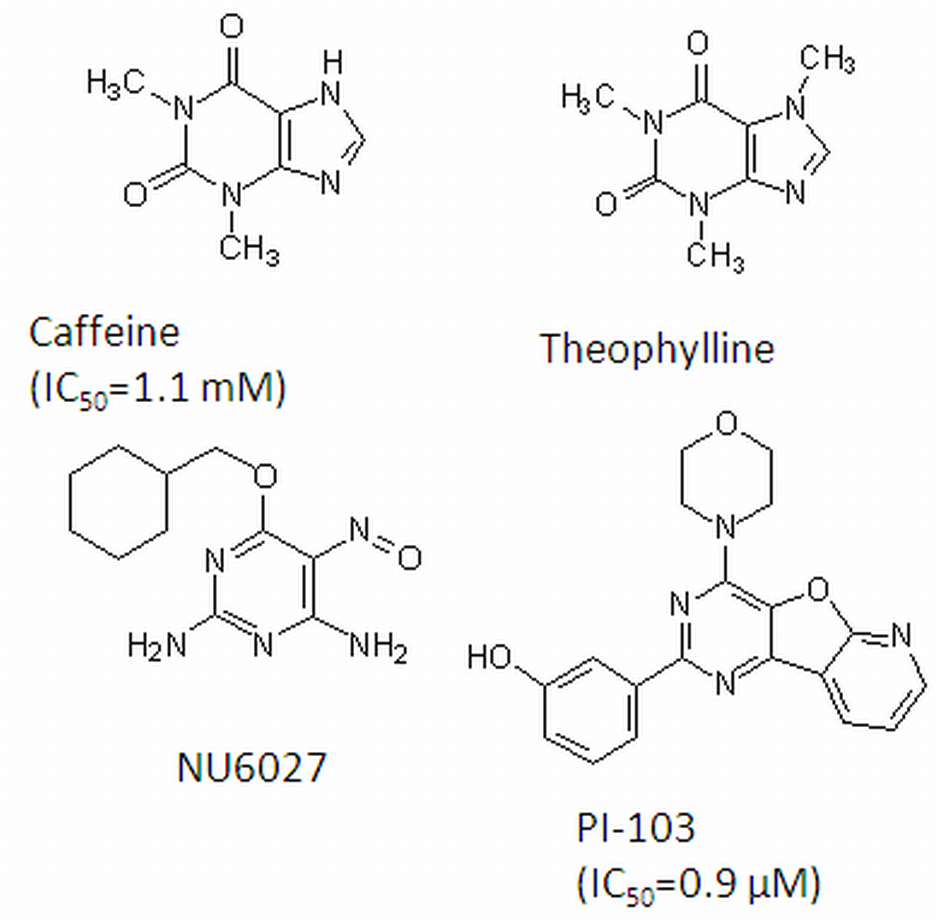


**Supplementary Figure 2**


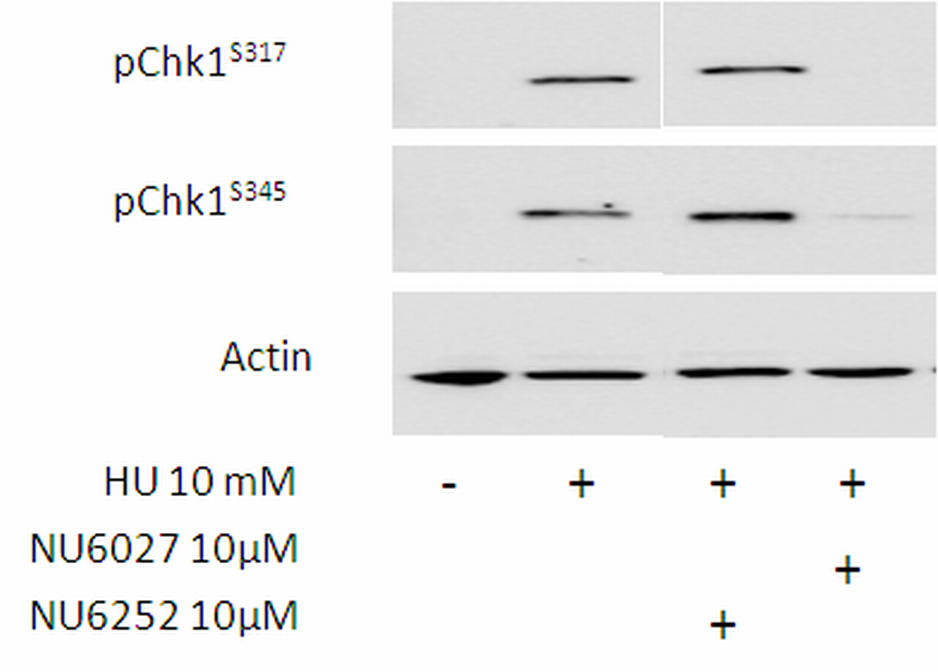


**Supplementary Figure 3**


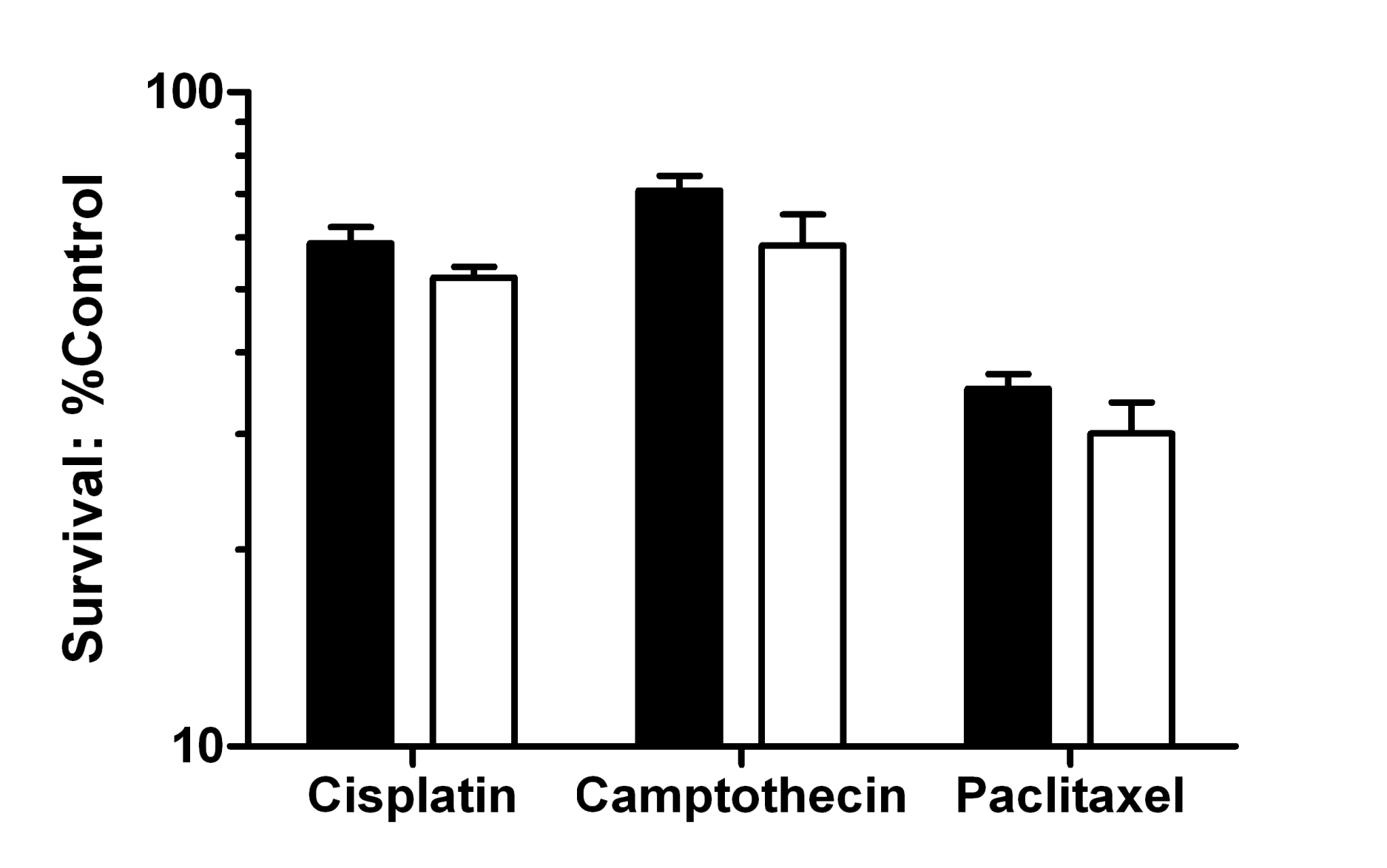


**Supplementary Figure 4**


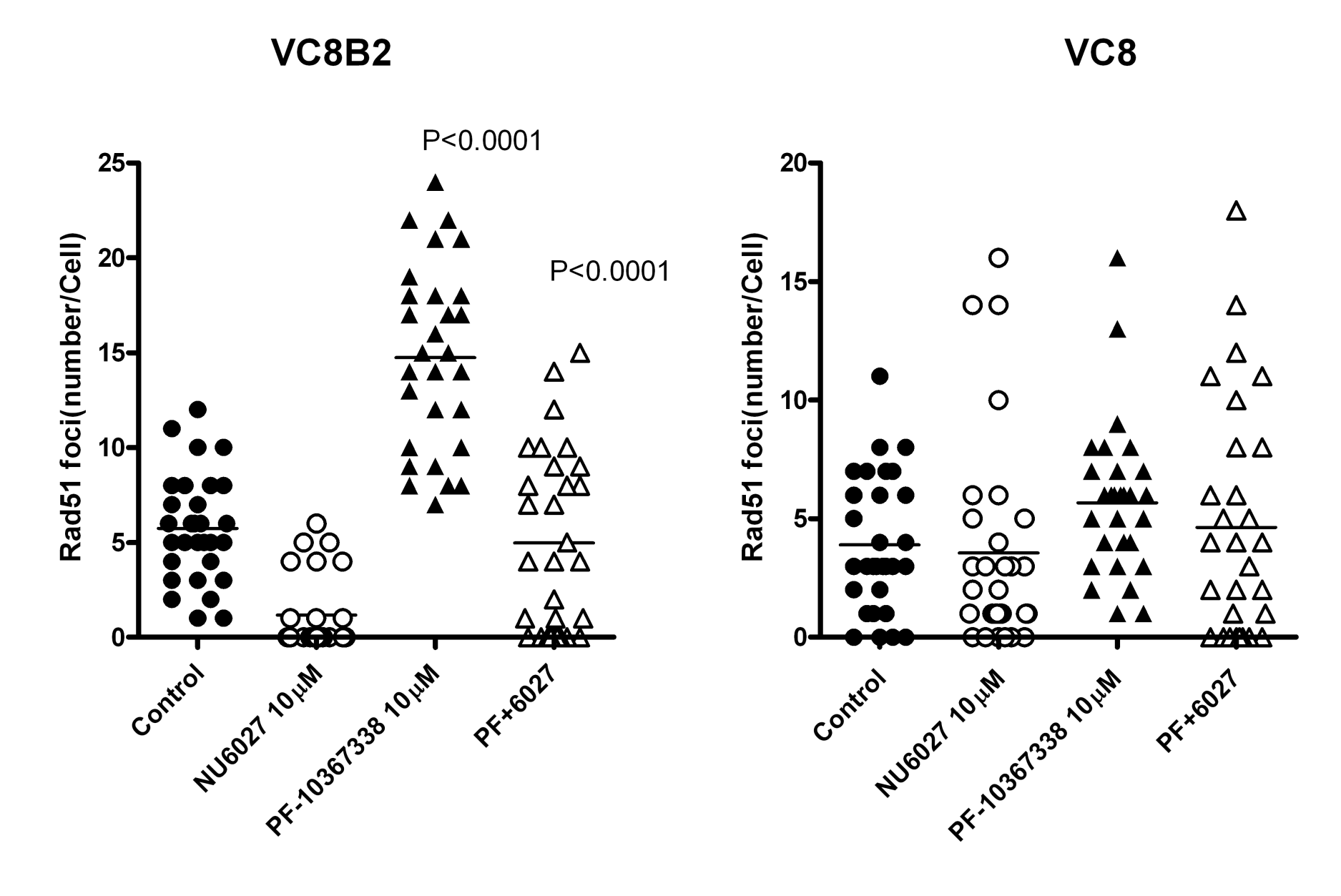

Supplement: Supplementary Information [file bjc2011243x1.doc]
